# Medical Association of Central New York

**Published:** 1872-12

**Authors:** 


MEDICAL ASSOCIATION OF CENTRAL NEW YORK.—This Association met at Rochester, on the 17th of December, and was as usual full of interest. Through the kindness of the Secretary, Dr. T. L. Brinkerhoff, of Auburn, we shall be enabled shortly to lay before our readers some of the papers read at this meeting.

